# Electrophysiological Study of the Antinociception Produced by the Coapplication of (±)-CPP and Propentofylline in Monoarthritic Rats

**DOI:** 10.1155/2013/315626

**Published:** 2013-04-04

**Authors:** Claudio Laurido, José L. Martínez, Francisco Morales

**Affiliations:** ^1^Laboratory of Neurobiology, Department of Biology, Faculty of Chemistry and Biology, University of Santiago of Chile, Avenue Libertador B. O'Higgins 3363, Casilla 40, Correo 33, 9170022 Santiago, Chile; ^2^Vice Rectory of Research, Development and Innovation, University of Santiago of Chile, Avenue Libertador B. O'Higgins 3363, Casilla 40, Correo 33, 9170022 Santiago, Chile

## Abstract

The NMDA receptor is central in the generation and maintenance of chronic pain. This receptor has several sites of modulation. One is the glutamate recognition site that can be blocked by (±)-3-(2-carboxypiperazin-yl)propyl-1-phosphoric acid or (±)-CPP. We investigated whether the effect of glial inhibition produced by propentophylline (PPF) can be enhanced when combined with (±)-CPP. We used Sprague-Dawley rats with experimental monoarthritis, administering intrathecally the ED_30_ for both drugs (3.97 *μ*g of (±)-CPP and 1.42 *μ*g of PPF), since this combination produces an antinociceptive supra-additive effect when used in mechanical nociception (Randall-Selitto test). The combination of (±)-PPF and CPP produced an antinociceptive effect which was greater than that each drug alone as tested by both the C reflex and windup. We conclude that the antinociceptive effect of the combination of (±)-PPF and CPP possibly generates a supra additive interaction type in monoarthritic rats.

## 1. Introduction

Pain continues to be a clinical problem difficult to solve for a significant proportion of patients due to the incomplete knowledge we have about the adaptive changes that occur in the neural substrates of the nociceptive system and glial cells in response to episodes of persistent pain. These changes primarily are associated with chronic inflammation processes or injury to peripheral and central nerves. In a chronic inflammatory process, tissue damage induces a persistent stimulation of nociceptors, which are peripheral nerve endings of primary afferent fibers responsible for pain transmission. Chronic activation of nociceptors by different chemical mediators induces hypersensitivity or nociceptive sensitization, which is reflected in changes in the basal activation levels of neurons and altered gene transcription (plasticity). This allows the appearance of hyperalgesia or an exaggerated response to a nociceptive stimulus and allodynia, or a nociceptive response against an innocuous stimulus [1]. Great advances in this field occurred in the 1980's when two groups demonstrated that NMDA receptor antagonists inhibit the hyperexcitability of nociceptive neurons in the spinal cord induced by stimulation of C fibers [2, 3].

As mentioned above, the NMDA receptor is important in the establishment of chronic pain. However, today we know other factors that may modulate this pain, such as glial cells [4]. In the last decade numerous studies have shown that glial cells of the spinal cord have close contact with neurons, and this led to the proposed term tripartite synapses [5]. This process contributes to synaptic modulation of neuronal excitability and synaptic transmission by increasing nociception transmission and thus the persistence of chronic pain. It has been shown that astrocytes and microglia in the dorsal horn of the spinal cord are active under a variety of conditions that cause chronic pain and hyperalgesia, such as inflammation due to the subcutaneous administration of inactivated mycobacteria [6] and peripheral nerve trauma [7], among others [8]. Because NMDA receptors and glia have an important role in the pathophysiology of chronic pain, we propose to evaluate if the coadministration of (±)-CPP and PPF could enhance the analgesic effect of each drug on chronic inflammatory pain. Similar results have been published in our laboratory, but using a behavioral test of paw pressure “technique” (Randall-Selitto) [9]. The utilization of electrical nociception (C reflex and windup) allowed us to test the drugs by a stronger procedure, since this nociceptive test is more demanding than mechanical nociception. The ultimate goal of drug combination is to obtain effective analgesia with a reduction in the incidence and severity of side effects, a fact that can be achieved by using lower doses of the drugs.

## 2. Methods

### 2.1. Animals

The experiments were done in male Sprague-Dawley adult rats weighting 250–300 g, both normal and monoarthritic. The experiments were run in accordance with the Universidad de Santiago de Chile Ethical Committee and the Ethical Guidelines for Investigations of Experimental Pain in Conscious Animals [10]. In order to minimize unnecessary suffering to the animals, a maximum of 5 animals were utilized in each experiment. Also, all the animals were submitted to a supervision protocol as described by Morton and Griffiths [11]. Briefly, this protocol allows us to quantify the pain (nociception) caused by an experimental procedure. It consists of five variables and each animal is scored. If the scores go above a certain number, animals have to be euthanized or the procedure has to be stopped immediately. Immediately after finishing an experimental procedure, the animals were euthanized with an overdose of urethane. 

### 2.2. Induction of Monoarthritis

Monoarthritic rats were used as a model of chronic inflammatory pain. Monoarthritis was induced in rats of 120 to 150 g by the method described by Butler et al. [12]. In brief, rats were inoculated with a volume of 50 *μ*L of Freund's adjuvant, in the right ankle joint. The adjuvant consisted of a solution of 60 mg of *Mycobacterium butiricum*, 6 mL of mineral oil, 4 mL of sodium chloride (0.9%), and 1 mL of Tween 80. Subsequently, this mixture was autoclaved at 120°C for 20 min and stored at room temperature until use. The injection of adjuvant produces a localized arthritic syndrome that becomes stable around the fourth week after-inoculation and establishes a persistent pain with hyperalgesia of the tibiotarsal joint which is maintained for a period exceeding two months. Monoarthritic rats were used between the 4th to the 5th week after induction of monoarthritis. Around 90%–95% of the injected rats developed mechanical hyperalgesia. 

### 2.3. Electrophysiological Determinations

Normal and monoarthritic rats were anesthetized with an intraperitoneal injection of 30% urethane dissolved in saline and then submitted to the C reflex and wind-up paradigm. The C reflex and windup are obtained by electrical stimulation by means of supramaximal (meaning a stimulus able to produce an electromyographic response) electric shocks applied subcutaneously to the fourth and fifth toes of the hind paw, territory innervated by the sural nerve, using two stainless steel electrodes. The stimulation was performed with a pulsed stimulation of 2 ms duration and a frequency of 1 or 0.1 Hz depending on the experiment. After 20 minutes of stimulation at 0.1 Hz to stabilize the response, the threshold current was determined (6.3 ± 0.4 mA for normal rats and 3.7 ± 0.6 mA for monoarthritic rats, *n* = 8 animals) and then twice the threshold intensity (meaning a current that elicits an electromyographic response in the 50% of the cases) was maintained throughout the experiment at 0.1 Hz, constituting the C reflex response. This was evidenced by electromagnetic activity registered from the biceps femoris ipsilateral [13]. When assessing the wind-up response the frequency of stimulation was raised to 1 Hz. Electromyographic recording was taken in a time window between 150 and 450 ms after the stimulus, in order to exclude the *A*-*δ* fibers response and then digitalized. Then, the digitalized response was processed using the CHART software v5.0. The recordings were made before and after administration of saline, (±) CPP, PPF, or both. For the C reflex and windup, records were taken at 5, 15, and 30 min. In the case of the C reflex, the values obtained were the average of the first 10 responses, while for the windup, we used the slope obtained for the first 7 recordings showing an increment in the response, calculated from the absolute value of the integrated response of the electromyogram (expressed in Volt per second). This C fiber activated reflex is equivalent to the R-III reflex recorded in man, representing a direct proportionality among subjective pain perception and the electromyographic intensity.

Results were expressed as the area under the curve (AUC) and then the groups were statistically compared. 

### 2.4. Statistics

Statistical analysis of the data was performed by analysis of variance (ANOVA) for the C reflex and windup. For all outcomes, the significance level was set at *P* < 0.05 and plotted as follows: *P* < 0.01 = ∗∗ and *P* < 0.001 = ∗∗∗. Results were expressed as mean percentage of the antinociceptive effect ± standard error (SE) for each experimental group versus baseline obtained before the injection of serum or of each of the drugs under study, as appropriate.

### 2.5. Intrathecal Injections

(±)-CPP (Tocris bioscience) was administered in single doses of 3.97 µg. PPF (Sigma) was administered in repeated doses of 1.42 µg/10 µL, once daily for a period of 10 days. The two drugs were administered via intrathecal (i.t.) injection in a volume of 10 *μ*L dissolved in saline; i.t. injection consisted of the administration of the drugs into the subarachnoid space between lumbar vertebrae L5 and L6 [14], using a Hamilton syringe with a needle 26Gx1/2′′. Entry into the subarachnoid space was evidenced by a slight movement in the tail of the rat as a result of the mechanical stimulation of the needle penetrating the meninges of the spinal cord. The daily PPF i.t. injection was done under brief halothane anesthesia (2 minutes at 96 : 4, oxygen : halothane, in percent). No sign of motor impairment was found in the rats submitted to these intrathecal injections as revealed by behavioral observations.

### 2.6. Experimental Groups

To evaluate the antinociceptive effect of both drugs in monoarthritic rats individually and in combination, the C reflex and windup were utilized. The animals were separated in the first stage of the study into 2 groups:

(1) intrathecal administration of (±)-CPP: 3.97 *μ*g/10 *μ*L (*n* = 5),
(2) daily i.t. PPF administration of 1.42 µg/10 µL (*n* = 5) for 10 days,
(3) daily administration of PPF at 1.42 µg/10 µL (*n* = 5) for 10 days. Then, at day eleven an i.t. administration of (±)-CPP: 3.97 *μ*g/10 *μ*L (*n* = 5).

Controls were provided by normal and monoarthritic rats receiving saline, as follows:

(1) normal group of the same age as the monoarthritic rats, receiving i.t. injection of saline instead of (±)-CPP, before testing (*n* = 5),
(2) monoarthritic saline group, receiving i.t. daily injection of saline for a period of 10 days followed by an i.t. injection of saline at day eleven, or a single injection at day eleven (*n* = 5).

## 3. Results

### 3.1. Nociception in Normal Rats: Area under the Curve of PPF and (±)-CPP Alone or in Combination in the Responses to the C Reflex and Wind-Up Test

**Figure 1:**
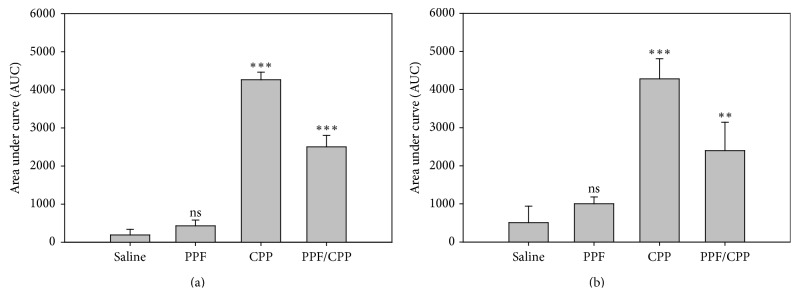
(a) Area under curve (AUC) of the antinociceptive effect in the C reflex for the ED_30_ of PPF for 10 days, ED_30_ of (±)-CPP, and the combination PPF/(±)-CPP compared to the saline administration in normal rats. It can be seen that the effect of PPF is nonsignificant with respect to saline (ns) indicating the null effect of this glial inhibitor. (b) Area under curve of the antinociceptive effect of the spinal windup for the ED_30_ of PPF for 10 days, ED_30_ of (±)-CPP, and the combination PPF/(±)-CPP compared to the saline administration in normal rats. Again, there was no effect of PPF in the windup. *P* < 0.01 = ∗∗, *P* < 0.001 = ∗∗∗, and ns = non significant.

The administration of the ED_30_ PPF for 10 days did not produce a significant change on the area under the curve (AUC) compared to saline control (Figure 1(a)). For saline, the AUC value was 191.8 ± 146 (Mean ± SEM) and for PPF was 432 ± 151. The i.t. injection of the ED_30_ of (±)-CPP resulted in a significant increase in the antinociceptive activity, being 22 times greater than the saline control group in the C reflex (AUC for (±)-CPP was 4265 ± 200). Finally, the i.t. injection of the effective doses of both combined drugs produced an antinociceptive activity 13 times greater than controls in the C reflex (AUC for PPF/(±)-CPP was 2504 ± 300). The effect of PPF in the windup was nonsignificant (Figure 1(b)). For saline, the AUC value was 508 ± 432 and for PPF was 1003 ± 179. The i.t. injection of the ED_30_ (±)-CPP was the biggest response (AUC = 4281 ± 529) being of approximately the same magnitude of (±)-CPP from C reflex in normal rats from Figure 1(a). Nevertheless, for either normal and monoarthritic rats, the administration of both drugs did not show an additive effect; rather, the response was located in between (the AUC value for the combination of PPF and (±)-CPP was 2398 ± 745).

### 3.2. Nociception in Monoarthritic Rats: Area under the Curve of PPF and (±)-CPP Alone or in Combination in the C Reflex and Wind-Up Test

**Figure 2:**
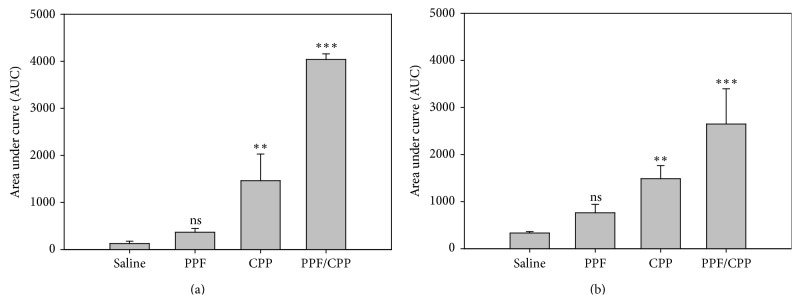
(a) Area under curve (AUC) of the antinociceptive effect in the C reflex for the ED_30_ of PPF for 10 days, ED_30_ of (±)-CPP, and the combination PPF/(±)-CPP compared to the saline administration in monoarthritic rats. (b) Area under curve of the antinociceptive effect of the spinal windup for the ED_30_ of PPF for 10 days, ED_30_ of (±)-CPP, and the combination PPF/(±)-CPP compared to the saline administration in monoarthritic rats. *P* < 0.01 = ∗∗, *P* < 0.001 = ∗∗∗, and ns = nonsignificant.

Figure 2(a) shows the absence of effect to the application of the ED_30_ i.t. PPF. For saline, the AUC value was 127.1 ± 50 (Mean ± SEM) and for PPF was 369 ± 77. (±)-CPP increases the AUC 11.5 times in respect to saline (AUC for (±)-CPP was 1464 ± 565). The application of the ED_30_ for the combination of both drugs resulted in an increase in the antinociceptive activity of around 32 times in respect to saline (AUC for PPF/(±)-CPP was 4037 ± 119). This represents a clear increment of antinociception with respect to the sum of the effects of the drugs separately, indicating a clear supra additive effect. The effect of PPF in the windup was nonsignificant (Figure 1(b)). For saline, the AUC value was 334 ± 30 and for PPF was 763 ± 179. The i.t. injection of the ED_30_ (±)-CPP showed an AUC = 1488 ± 277, being of approximately the same magnitude of (±)-CPP from C reflex in monoarthritic rats from Figure 2(a). The application of the ED_30_ for the combination of both drugs resulted in an increase in the antinociceptive activity of 117% with respect to the sum of the effects of each drug separately, (AUC for PPF/(±)-CPP was 2649 ± 748), indicating a more modest supra additive effect.

## 4. Discussion 

Results obtained in this study show that there exists an analgesic effect when combining a glial inhibitor (PPF) and an NMDA-receptor antagonist ((±)-CPP) in both normal and monoarthritic rats using the C reflex and wind-up paradigm. PPF [15] has inhibitory effects on activity of phosphodiesterase type I, II, and IV and on adenosine extracellular transporters in glial cells [8], thereby modifying intracellular cyclic nucleotide homeostasis leading to a decrease of the production of proinflammatory cytokines and free radicals in these cells. As mentioned before, we used the ED_30_ values obtained from the Randall-Selitto determinations from Morales et al. (2012) [9]. 

 There was no effect of PPF alone in both normal and monoarthritic rats. For normal rats, one would not expect that PPF produces antinociception, since PPF is only able to act on activated glial cells, generated by a neuronal lesion or chronic pain [9]. Nevertheless, this apparent absence of PPF effect in monoarthritic rats deserves some attention. The value of 1.42 µg/10 µL PPF administrated daily for ten days is rather low and may be not enough to completely inhibit the glial cells. This particular series of experiment was designed on purpose. The idea was to use the minimal PPF concentration that in combination presents an effect, modest in normal rats, but important in monoarthritic rats, without the saturating effects that might obscure the results if PPF concentration would have been greater. In monoarthritic rats, the effects of the combined drugs are possibly supra additive. Briefly, a supra additive effect for two or more drugs implies that the sum of the effects produced by the drugs alone is lower than the effects produced by the combination. Nevertheless, as pointed by Chou [16], in order to have a supra additive effect of drugs, it is necessary that the mechanisms of action of both drugs are at least “partially independent,” situation in agreement with PPF and (±)-CPP, one acting as a glial inhibitor and the other directly blocking the NMDA glutamate recognition site. 

In normal rats, the results indicate that (±)-CPP presents an antinociceptive effect for both the C reflex and windup. This appears to be unexpected, since the NMDA receptor should not be active under acute pain conditions. But there is abundant evidence in the literature indicating that the NMDA receptors are active in acute pain conditions [9, 17]. Also, this effect was tested on three different nociceptive tests: tail-flick, hot plate, and formalin, indicating that NMDA receptors may be involved “in functionally divergent nociceptive systems” [18], but this is not necessarily in contradiction with the role of the NMDA receptor on the establishment and persistence of chronic pain episodes. In this case, two major mechanisms appear to contribute to the resultant of this increased synaptic efficacy: (1) alterations in ion channels (Kv4.2 K^+^ channels) and receptor activity (NMDA and (2-amino-3-(5-methyl-3-oxo-1,2-oxazol-4-yl)propanoic acid) (AMPA) receptors) and (2) trafficking of AMPA receptors to the membrane. Both events are due to phosphorylation by protein kinases, thereby increasing synaptic efficacy by altering channel open time, increasing bursting, removing the Mg^2+^ channel blockade, and promoting trafficking of receptors to the synaptic membrane [19, 20]. These mechanisms clearly represent a positive intracellular feedback loop, whereby membrane receptor activation by pronociceptive neurotransmitters leads to increased activity of the same receptors via phosphorylation by protein kinases. A second positive, but extracellular, feedback loop is represented by the products of the CaMKII-phosphorylated enzymes nitric oxide (NO) synthase and cyclooxygenase-2, the diffusible messengers NO and prostaglandin E_2_, which retrogradely diffuse to presynaptic nociceptive axon terminals and increase neurotransmitter release. Nevertheless, the effect of (±)-CPP and PPF in normal rats is not additive; the combination of both drugs results in a response located in between (±)-CPP and PPF antinociception. In monoarthritic rats, for C reflex and windup, on the contrary, there was a supra additive response. As discussed earlier, even though the effect of PPF was not statistically significant for C reflex and windup, the resultant AUC for the combination of (±)-CPP and PPF showed an increment in antinociception bigger than the effect of the drugs alone. For the C reflex, the AUC combination of PPF and (±)-CPP was higher than the sum of the effects of PPF and (±)-CPP alone. For the windup, the values were more modest, being only 70% higher than the sum of the AUC of the PPF and (±)-CPP alone. This result is not surprising, since PPF and (±)-CPP have to act on a monoarthritic condition, were glial proinflammatory cytokines (interleukin-1*β*, interleukin-6, and tumor necrosis factor, among others) are overexpressed, requiring higher doses of PPF to act effectively. 

## 5. Conclusion

We show for the first time that the glial inhibitor PPF can synergistically enhance the effect of (±)-CPP, a drug that inhibits the activity of the NMDA receptor in the C reflex and spinal windup. This contribution opens a field of the association of glial inhibitors and NMDA receptor blockers for the treatment of chronic pain episodes.
